# Health Tract, No. 272

**Published:** 1868-03

**Authors:** 


Hall's Journal of Health, March, 1868.

HEALTH TRACT, No. 272.

### EATING MATHEMATICALLY.

Persons in good health should not eat any article of food simply because it is "healthy," nor avoid an article because some one says it is "unhealthy;" nature's instincts are a better and safer guide, for she craves food, the distinctive elements of which are needed in the system; hence no man's likes or dislikes of an article of diet should be the guide of another, any more than all soils should require the same fertilizer, in quality and quantity.

Sometimes indeed, but rarely in good health, a man may crave earnestly an article of food, and after eating it, feel uncomfortable; yet, rather than conclude it did not agree with him and discard it, a smaller quantity should be taken next time; and very often that smaller quantity, well divided, prepared properly and eaten slowly, will "agree," simply because the system needed only that smaller quantity.

Brown Bread is said to be "good for" many persons, by its keeping the system open and free; but if a man is well enough in that respect, he would do well not to eat brown bread, unless he was fond of it, so as to have it to fall back upon, should he need its medicinal effect. In short, eat according to the natural appetite as to quantity and quality, and not according to artificial rules and regulations.

If a man is an invalid and has a family physician, it is safer and better to put himself under that physician's guidance; if he has no physician, let him feel his own way, taking small quantities at regular intervals, and closely observe the effects. But for both sick and well, it is just as unwise to measure and weigh each meal day after day, as it would be to wear the same amount of clothing, and consume the same amount of fuel every day in the year, winter and summer. In mature life, we eat for two reasons, to repair wastes and to keep the body warm; the wastes are in proportion to the preceding exercise, and the internal warmth needed is in proportion to the temperature of the atmosphere about the body. If you eat to-day, while idle and the thermometer is at sixty, as much as you did yesterday when it was at zero and you worked hard, you will certainly be sick to-morrow. After all, don't make a god of your belly, but accustom yourself to think of eating, and what you shall eat, only when the time for eating comes; a beast or a glutton may do otherwise, a man will not.

